# Supplementary material for: Central Nervous System Disorders with Auto-Antibodies in People Living with HIV
Source: Microorganisms. 2024 Aug 24;12(9):1758. doi: 10.3390/microorganisms12091758 (PMC11433650; doi:10.3390/microorganisms12091758)
Supplement: Supplementary file 1 [file microorganisms-12-01758-s001.zip › microorganisms-3097130-supplementary.pdf]

Patient	MRI lesions	Opportunistic infections	Other infections
**1**	Myelitis D3-D9 with hyperintensity and enhancement	None	None
**2**	Normal, nonspecific	Candida (oral thrush)	Previous Neurolue
**3**	Bilateral frontotemporal inflammatory lesions resembling encephalitis, with mass effect; demyelinating foci	Candida (oral thrush)	None
**4**	White matter involvement: demyelinating, cortico-subcortical leukoencephalopathy; bilateral frontoparietal, insular with swelling appearance	Candida (oral thrush); PML-IRIS	None
**5**	Encephalitis-like lesions with demyelinating component affecting the brainstem and mesial temporal lobes with swelling	None	None
**6**	Normal, nonspecific	Candida (oral thrush)	None
**7**	Bilateral cerebellar demyelinating lesions	PML; Candida esophagitis	Previous labial HSV; HBV (HBeAg negative chronic infection, non-hepatitis)
**8**	Inflammatory foci in the frontobasal regions affecting the straight gyri and the anteroinferior portion of the cingulate gyri, deep right frontal focus, and left frontoparietal cortico-subcortical focus	None	HBV (HBeAg negative chronic infection, non-hepatitis); previous primary syphilis (treated, negative at subsequent controls)
**9**	White matter hyperintensities, likely subcortical vascular impairment; inflammatory foci	None	HCV (treated)
**10**	Hyperintensity of the left lateral ventricle anterior horn with perilesional edema and compression of the ipsilateral ventricle	PCNSL; Candida esophagitis	Previous labial HSV
**11**	Callosal atrophy	None	None
**12**	Normal, nonspecific	None	None

Supplementary Table S1: the table describes additional clinical and radiological characteristics of patients included in the analysis**.** HBV: hepatitis B virus; HBeAg: hepatitis B e antigen; HCV: hepatitis C virus; HSV: herpes simplex virus; PML: progressive multifocal leukoencephalopathy ; IRIS: immune reconstitution inflammatory syndrome; PCNSL: primary central nervous system lymphoma; D: dorsal.
